# The 100th: An appealing new species of *Dendropsophus* (Amphibia: Anura: Hylidae) from northeastern Brazil

**DOI:** 10.1371/journal.pone.0171678

**Published:** 2017-03-08

**Authors:** Iuri Ribeiro Dias, Célio Fernando Baptista Haddad, Antônio Jorge Suzart Argôlo, Victor Goyannes Dill Orrico

**Affiliations:** 1 Departamento de Ciências Biológicas, Universidade Estadual de Santa Cruz, Ilhéus, Bahia, Brazil; 2 Programa de Pós-Graduação em Zoologia, Universidade Estadual de Santa Cruz, Ilhéus, Bahia, Brazil; 3 Universidade Estadual Paulista, Departamento de Zoologia, Instituto de Biociências and Centro de Aquicultura (CAUNESP), Campus Rio Claro, São Paulo, Brazil; Universita degli Studi di Roma La Sapienza, ITALY

## Abstract

We describe a new species of the *Dendropsophus leucophyllatus* Group from the Atlantic Forest of the southern region of State of Bahia, Brazil. It can be distinguished from all species of the *D*. *leucophyllatus* Group on the basis of morphological characters (especially its unique dorsal pattern and snout in dorsal view), advertisement calls and divergence in mitochondrial DNA gene sequences. The inclusion of *D*. *anceps* on the group remains controversial but our phylogenetic analyses do not recover the new species as sister to syntopic species of the *D*. *leucophyllatus* Group (with or without *D*. *anceps*). These results also highlight the palimpsest that is past relation between the Atlantic and Amazon forests.

## Introduction

The southern portion of the State of Bahia, Brazil (the “Hiléia Bahiana”, see [1]) harbors a unique and diverse amphibian fauna [2,3] and recently, many new species have been described from localities therein (e.g. [4,5]). Several of these are phylogenetically closer to Amazonian species than to other species of the Atlantic Forest (e.g. [6,7,8]). Although *Dendropsophus* is speciose not many species have been described lately (see [9,10–12]), especially for the Atlantic Forest [13,14].

The genus includes today 99 species [14,15], divided in nine groups according to Faivovich et al. [16]. One of these groups—the *Dendropsophus leucophyllatus* Group, or “leaf-gluing treefrogs” (see [17])—has been receiving much taxonomic and systematic attention, including the description of three new species in recent years [10,11,17]. The monophyly of this group is disputed and the placement of *D*. *anceps* in the group is controversial (see [10], and [11] for a discussion). If the monophyly of the group including *D*. *anceps*, is corroborated, the presence of two glandular pectoral patches (see [16,18,19]) seems the best candidate morphological synapomorphy for the group [10]. Nevertheless, only male *D*. *anceps* present such patches while both sexes of other species of the group present them—therefore, the putative synapomorphy should be presence of those patches in males (see [10]).

Today, 11 species are assigned to the *D*. *leucophyllatus* Group, but only two (*D*. *elegans* and *D*. *anceps* considering the latter as a member of the group) occur in the Atlantic Forest [10,15]. One species (*D*. *ebraccatus*) dwells in Central America and the remaining species occur through the Amazon biome [11,15,20,21].

In this paper, on the basis of morphology, bioacoustics and molecular data, we describe a new species of the *D*. *leucophyllatus* Group from southern State Bahia, Brazil. This is the third species of the group associated with the Atlantic rainforest and—at the moment—the 100^th^ valid species assigned to the genus.

## Materials and methods

### Ethics statement

This study was conducted with appropriate permissions and guidelines from the responsible authority—licence #13708 from “Instituto Chico Mendes de Conservação da Biodiversidade” (ICMBio) that also evaluates protocols for our collection and research. Muscle samples were taken from thighs and stored in absolute ethanol for subsequent DNA extraction and sequencing. Collected specimens were not recognized as belonging to threatened species and they are not listed in IUCN Redlist or by CITES.

**Fig 1:**
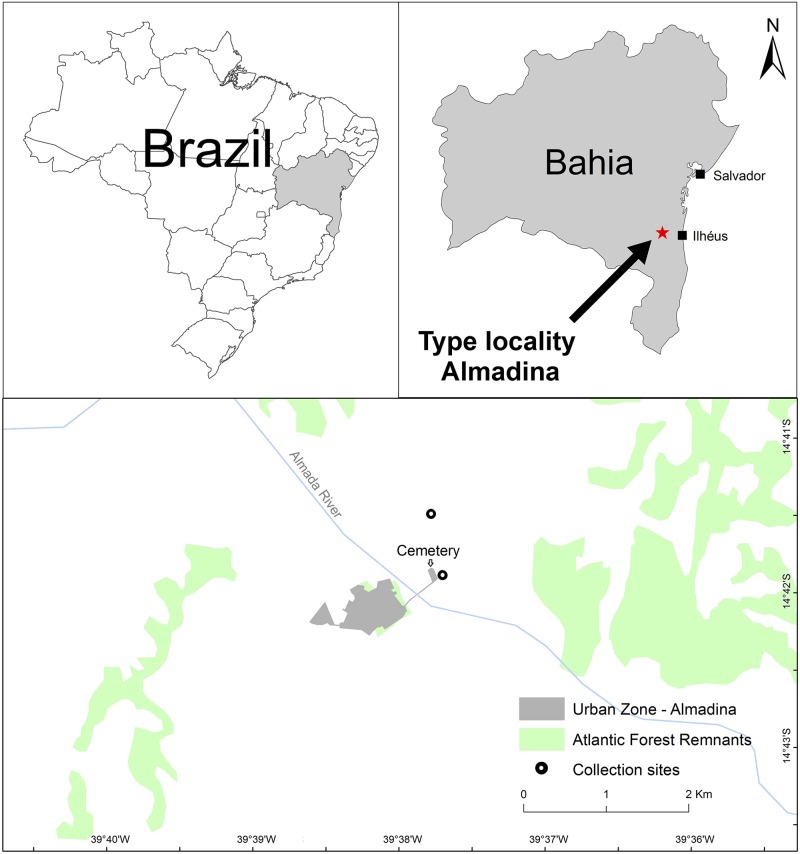
Geographic distribution of *Dendropsophus nekronastes* sp. nov. Squares = reference cities in the Bahia state; star = type locality; circles = collection sites within municipality of Almadina.

The specimens were collected by hand at the municipality of Almadina, State of Bahia, northeastern Brazil (Fig 1) in two localities—Almadina city cemetery (14° 42' 0.51" S, 39° 37' 48" W; 303 m a.s.l.) and “Fazenda Rovil” (14° 41' 37" S, 39° 37' 54 " W; 311 m a.s.l.). Specimens were euthanized with 5% lidocaine (acting as the primary method of euthanasia), fixed in 10% formaldehyde, and subsequently preserved in 70% ethanol.

### Taxa and specimens

Specimens used in the description or examined for comparisons are listed on S1 Appendix and are housed in the following collections: Museu de Zoologia da Universidade de Santa Cruz, Ilhéus, BA, Brazil (MZUESC); Museu de Zoologia da Universidade de São Paulo, São Paulo, SP, Brazil (MZUSP); Célio F. B. Haddad Collection, Universidade Estadual Paulista, Rio Claro, SP, Brazil (CFBH); MCNAM (Museu de Ciências Naturais, Pontifícia Universidade Católica de Minas Gerais, Brazil); MHUA-A (Museo de Herpetología Universidad de Antioquia, Colombia); QCAZ (Museo de Zoología, Pontificia Universidad Católica del Ecuador); USNM (National Museum of Natural History, Smithsonian Institution, USA).

### Morphological and bioacustic measurements and analyses

Measurements are in millimeters and follow Duellman [19,21] of Napoli and Caramaschi [22]—when landmarks differ between these two contributions, we explain which were used. Abbreviations are SVL (snout—vent length); HL (head length); HW (head width); ED (horizontal eye diameter); TYD (horizontal tympanum diameter); IND (internarial distance); IOD (interorbital distance); EN (eye—nostril distance); NSD (nostril—tip of snout distance); THL (thigh length); TL (shank length); FL (foot length, from the proximal edge of the inner metatarsal tubercle to the tip of Toe IV); TAL (tarsus length, from the heel to the proximal edge of the inner metatarsal tubercle); 4TD (fourth toe disc diameter); DF3 (width of disc on third finger); HAL (hand length, from the proximal edge of the thenar tubercle to the tip of Finger III); and FOL (forearm length, from the tip of the elbow to the proximal edge of the thenar tubercle). We measured SVL, HL, HW, THL, TL and FL with digital callipers to the nearest 0.1 mm. The remaining measurements were taken under a stereomicroscope equipped with an ocular micrometer. Webbing formula follows the standards of Savage and Heyer [23,24] with the modifications of Myers and Duellman [25].

We recorded the advertisement calls of two males (MZUESC 10175; SVL = 26.6 mm and MZUESC 10176; SVL = 28.5 mm) of the new species with a Sennheiser ME45 unidirectional microphone attached to a Tascam DR1 digital recorder. In all cases, microphone was placed at a distance of 20 cm from the recorded specimen. Calls were analyzed using the Raven Pro 1.4 at 44.1 kHz with 16 bit resolution. Waveforms and audiospectrograms were produced with the following parameters: 256 points FFT, hann window, with 50% overlap. Advertisement call terminology follows Duellman and Trueb [26]. Exceptions are advertisement call type terminology that follows Heyer et al. [27] for “Type I” and “Type II” notes or Wells [28] for “primary” and “secondary” notes. Notice that “Type I” is synonym of “primary” and “Type II” of “secondary” for *Dendropsophus* advertisement calls [29]. Monophasic calls have a single type of note while biphasic calls, two (see [12]). We measured number of notes, call duration (s), number of pulses per call per note, duration (s) of Type I and Type II notes, number of pulses per Type I and per Type II note, Type I and Type II note pulse rate, Call pulse repetition rate, Call repetition rate per minute, and dominant frequency (Hz).

### Sequencing and phylogenetic analyses

To corroborate our new species as a member of the *D*. *leucophyllatus* Group, we sequenced the complete 12S rRNA gene, a fragment of the 16S rRNA gene, and the intervening valine-tRNA (totalizing 2633 bp) of two specimens of the new species (MZUESC 9979 and 10177; GenBank accession number: KY552470-KY552471) using the same primers and protocols for extraction, amplification, purification, sequencing, and sequence edition of Faivovich et al. [16]. We gathered all comparable mitochondrial sequences of *Dendropsophus* and selected closely related genera for outgroups from GenBank (see S2 Appendix) based on Faivovich et al. [16]. We excluded unnecessary duplicates (e.g. many *D*. *minutus* specimens) keeping the longest available sequences. In addition, we sequenced the same fragments from a syntopic individual of *D*. *elegans* (MZUESC 9968; GenBank accession number:KY552469) to confirm their distinctiveness.

Alignment was performed by MAFFT v7 [30] under the Q-INS-i strategy that is the most appropriate to our dataset according to program documentation. We avoided gathering nuclear data because our aim was solely to confirm our species as a member of the genus. Given that mitochondrial DNA is more variable than nuclear DNA, it seems a better choice to assess the relationships with closely related species. The resulting matrix was analyzed through Maximum Parsimony (MP) and Bayesian inference (BI) criteria; input files are available on S1 File (MP) and S2 File (BI).

The resulting matrix was analyzed with Maximum Parsimony (MP) under a New Tech approach as implemented in TNT [31], under equal weighting, using all settings, algorithms, and nodal support evaluation as employed by Rivera-Correa and Orrico [10]. Random seed was set to 0 (aleatory) and minimum length was found 100 times.

Bayesian inference was implemented in MrBayes 3.2.6 [32] and run on the CIPRES Science Gateway v3.3 [33]. The evolutionary model most appropriate for each gene was chosen using the Akaike information criteria, implemented in PartitionFinder 1.1.1 [34]. The GTR + I + G substitution model was selected as the optimal nucleotide substitution model for the data set. We then used a Bayesian inference search in a Markov Chain Monte Carlo analysis, run in two independent runs, each with four chains and sampling every 1000 generations for 80 million generations. An adequate burn-in (the first 20% trees were excluded) was determined by examining a plot of the likelihood scores of the heated chain for convergence and stationarity. Tracer 1.6 [35] was used to confirm the quality of the parameters of the analysis.

### Nomenclatural acts

The electronic edition of this article conforms to the requirements of the amended International Code of Zoological Nomenclature, and hence the new names contained herein are available under that Code from the electronic edition of this article. This published work and the nomenclatural acts it contains have been registered in ZooBank, the online registration system for the ICZN. The ZooBank LSIDs (Life Science Identifiers) can be resolved and the associated information viewed through any standard web browser by appending the LSID to the prefix “http://zoobank.org/”. The LSID for this publication is: urn:lsid:zoobank.org:pub:71D82C22-2D2B-4A79-B610-7941FBD0391B. The electronic edition of this work was published in a journal with an ISSN, and has been archived and is available from the following digital repositories: PubMed Central and LOCKSS.

## Results

### Description of new species

*Dendropsophus nekronastes* **sp. nov**.

urn:lsid:zoobank.org:act:2EFB97FF-AFF3-4C73-80BE-900A4F46E211

#### Holotype

**Fig 2:**
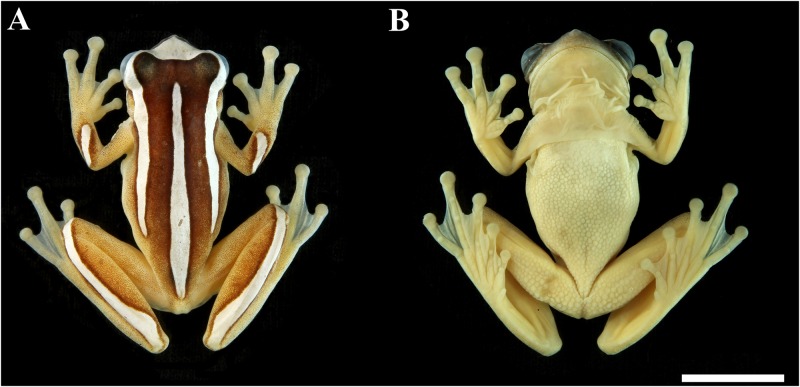
Holotype of *Dendropsophus nekronastes* sp. nov. (A) Dorsal and (B) ventral views (MZUESC 10178). Scale bar = 10 mm.

**Fig 3:**
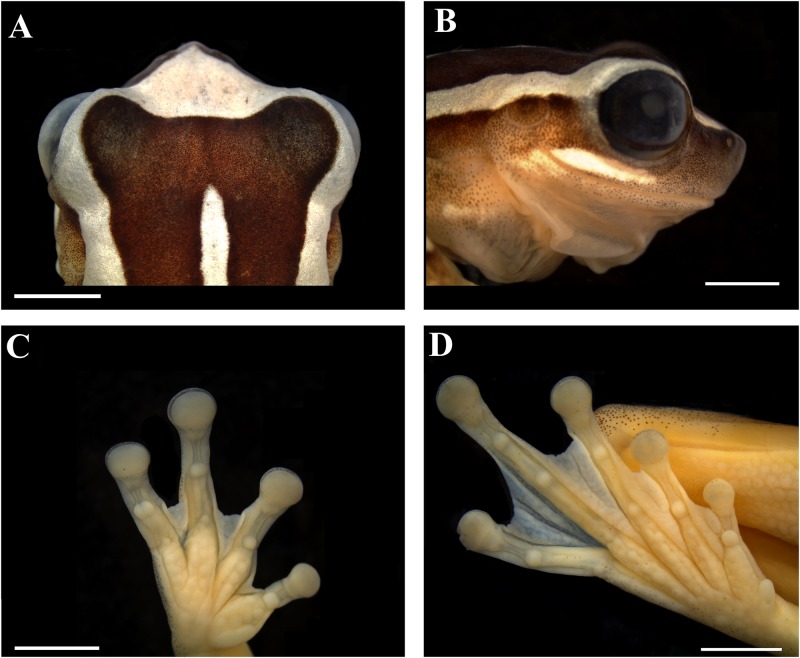
Holotype of *Dendropsophus nekronastes* sp. nov. (A) Dorsal and (B) lateral views of the head, (C) palmar and (D) plantar views (MZUESC 10178). Scale bars = 3 mm.

MZUESC 10178, adult male (Figs 2 and 3), collected in a pond near the Almadina city cemetery (14° 42' 0.51" S, 39° 37' 48" W; 303 m a.s.l.), municipality of Almadina, State of Bahia, Brazil, on 04 February 2012, by Iuri R. Dias.

#### Paratypes

MZUESC 10174–10177 (adult males); 10179–10184 (adult males), MZUESC 10185 (adult female), collected with the holotype; MZUESC 9979 (adult female), MZUESC 9980–9989 (adult males), collected in the same collection site of the holotype, on 17 December 2011, by Antônio J.S. Argôlo; MZUESC 10221–10223 (adult males) collected in the same municipality, on other pond, on a farm known as “Fazenda Rovil” (14° 41' 37" S, 39° 37' 54 " W; 311 m a.s.l.), on 05 February 2012, by Iuri R. Dias.

#### Diagnosis

We assign *Dendropsophus nekronastes* to the *D*. *leucophyllatus* Group based on the presence of the glandular pectoral patches—the putative synapomorphy of the group—and the results of the phylogenetic analysis indicate that new species is nested in the group. The species can be diagnosed by the following combination of character states: (I) SVL 24.1–28.9 mm in males (n = 24) and 31.8–35.4 mm in females (n = 2); (II) short snout (approximately 30% of HL), truncated in lateral view and mucronate in dorsal view; (III) tympanum rounded, tympanic ring distinct, except for the dorsal margin; (IV) *canthus rostralis* straight and loreal region nearly vertical; (V) dorsum dark brown with white markings consisting of a triangular head blotch connected to dorsolateral stripes that cover the eyelid and extend to the posterior third of body (composing an inverted U-shape, see Fig 2); (VI) a medial white line that extends from the interorbital area to the sacral region; (VII) wide white suborbital marking, as long as eye diameter; (VIII) two nearly transverse white bars in the forelimbs, one in the arm, from the arm insertion point to arm midpoint, and the other from the elbow to the wrist; (IX) shank with a regular dorsal white stripe covering nearly all its surface, from the knee to heel; (X) nuptial pads present, glandular, small, covering only the medial area above the prepollex; (XI) red iris, pupil black, elliptical, and horizontal; (XII) axillary membrane extending to the half of the arm (when positioned transversally to the body); (XIII) pectoral glands present, well-marked, oval, present in both males and females; (XIV) vomerine teeth present; and (XV) advertisement call composed of a series of 1–3 pulsed notes, average duration of 0.305 s and dominant frequency ranging between 4478.0–4823.4 Hz.

#### Holotype description

Adult male, SVL = 28.5 mm; head slightly wider then long (HW/HL = 1.02; HW 33.3% of SVL; HL 32.6% of SVL); snout short (EN/HL = 0.279; NSD/HL = 0.150), mucronate in dorsal view and truncated in lateral view; nares terminal and slightly protuberant, elliptical, opening anterolaterally; eyes large (ED/HL = 0.376) and prominent; tympanum nearly circular, being slightly larger than high; its diameter slightly smaller than width of discs on third finger (TYD/DF3 = 0.89) and of fourth toe (TYD/4TD = 0.94), being 2.18 times smaller than eye diameter; tympanic ring distinct, except for its upper quarter that is covered by the supratympanic fold; supratympanic fold extending from posterior corner of the eye to anterior insertion of arm. Forearm diameter slightly larger than arm diameter; axillary membrane present and reaching the middle of the arm; finger discs large and transversally elliptic; relative length of fingers II < III < V < IV; subarticular tubercles rounded; distal tubercle of Finger V bifid; supernumerary tubercles present, especially at palmar area; external metacarpal tubercle slightly visible and divided; no visible nuptial excrescences at prepollical area; glandular nuptial pads present, arranged in a thin line; internal metacarpal tubercle oblong; palmar webbing formula I2 — 2II1^+^– 1 ^1/2^III2–2^-^IV. Hindlimbs long, shank longer than thigh and their sum larger than SVL (TL/SVL = 0.564; THL/SVL = 0.519); toes relative length I < II < III < V < IV; plantar subarticular tubercles present and round; supernumerary plantar tubercles present, especially at plantar area; internal metatarsal tubercle oblong; external metatarsal tubercle slightly visible and divided; plantar webbing formula I0–2-II0 — 2III1 — 2IV2 – 0V. Cloacal opening covered by a cloacal sheath that reaches only the thighs dorsal third; skin smooth, except for belly and ventral proximal half of thighs that are rugose; two glandular patches visible on chest, separated from each other by about half their width). Vocal slits present, slit-shaped (see [29]). Vomerine teeth present, but not arranged in series; dentigerous processes not touching each other, located between choanae; tongue cordiform; vocal sac single, subgular, well developed, extending to the proximal half of arm.

#### Measurements of holotype (in mm)

SVL = 28.5; HL = 9.3; HW = 9.5; ED = 3.5; TYD = 1.6; IND = 2.5; IOD = 4.2; EN = 2.6; NSD = 1.4; THL = 14.8; TL = 16.1; FL = 13.8; TAL = 8.0; 4TD = 1.7; DF3 = 1.8; HAL = 9.3; FOL = 4.8

#### Color in life

**Fig 4:**
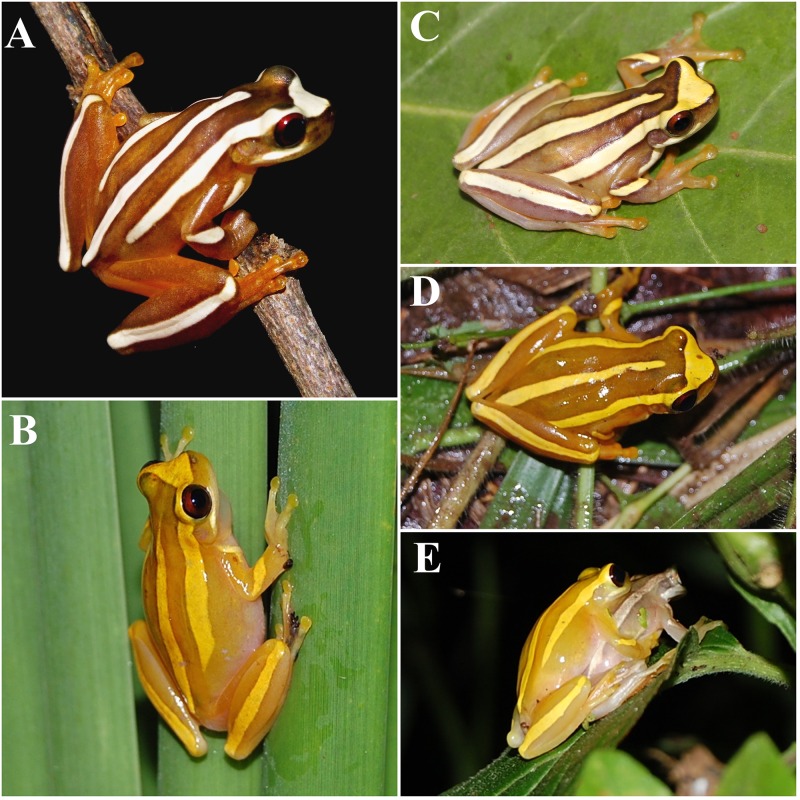
*Dendropsophus nekronastes* sp. nov. in life. (A, C) Color variation between day and (B, D, E) night. Vouchers: A) not identified; C) MZUESC 9979; D) MZUESC 9983; B) and E) not collected. Photos A) and C) Iuri R. Dias; B), D) and E) Juliana A. de Jesus.

*Dendropsophus nekronastes* specimens present small variation between day and night colorations. During day and night, the inverted U-shape, medial sacral line, suborbital bar, and limb stripes are yellowish-white; iris is dark red; the mental region and vocal sac are bright-yellow; the belly and chest are of an metallic white; the ventral surfaces of limbs are dark yellowish-orange. During the day, the dorsum background is dark brown, yellowish-white blotches are surrounded by dark caramel brown stripes, thighs are deep orange, and groin is bright yellow. At night, the dorsum background lightens to a dark yellow and dark caramel brown stripes surrounding the yellowish-white blotches become less evident; otherwise, similar to the day pattern (Fig 4).

#### Variation among paratypes

Overall, the type series is morphologically congruent with the holotype, although females are larger than males. Variation in measurements is presented in Table 1. The two females (MZUESC 9979 and 10185) present a small longitudinal stripe in the flanks not found in male specimens. The dorsolateral striped pattern is similar among all individuals; the width of the dorsolateral stripes is variable. In six specimens (MZUESC 10175, 10176, 9983, 9986, 10181 and 10155), dorsal coloration is lighter (more similar to night pattern). The medial sacral line is continuous in all individuals (except for MZUESC 9980 that presents a small interruption) and its anterior edge reaches anteriorly between the intertympanic to the interorbital levels. The forearm stripe varies in shape and length but always restricted to the forearm. Snout in lateral view varies between truncate (80%, N = 20) or slightly rounded (20%, N = 5) and in dorsal view, between mucronate (80.7%, N = 21) and truncated (19.3%, N = 5; MZUESC 9982; 9986; 10175; 10221; 10223).

**Table 1:** Measurements (mm) of adult specimens of *Dendropsophus nekronastes* sp. nov. (S.D. = standard deviation).

Measurements	Males (n = 24)				Females (n = 2)			
	Min	Max	Mean	S.D.	Min	Max	Mean	S.D.
**SVL**	24.1	28.9	27.3	1.0	31.8	35.4	33.6	2.5
**HL**	7.5	9.3	8.7	0.4	9.35	11.2	10.3	1.3
**HW**	7.8	9.5	8.9	0.3	9.7	10.7	10.2	0.7
**ED**	2.8	3.7	3.3	0.2	3.1	3.8	3.5	0.5
**TYD**	1.1	1.6	1.4	0.1	1.5	1.7	1.6	0.1
**IND**	2.0	3.0	2.4	0.2	2.6	3.8	3.2	0.8
**IOD**	3.5	4.2	3.8	0.2	4.2	4.3	4.3	0.1
**EN**	2.0	2.8	2.6	0.2	3	3.2	3.1	0.1
**NSD**	1.0	1.4	1.3	0.1	1.5	1.6	1.6	0.1
**THL**	12.7	15.0	14.1	0.6	15.8	18.35	17.1	1.8
**TL**	14.1	16.1	15.1	0.6	14.65	19.5	17.1	3.4
**FL**	12.0	13.9	13.0	0.5	15.1	17.7	16.4	1.8
**TAL**	7.0	8.4	7.7	0.3	8.0	9.7	8.8	1.2
**4TD**	1.1	1.7	1.4	0.2	1.5	2.1	1.8	0.4
**DF3**	1.1	1.8	1.5	0.2	1.6	2.4	2.0	0.6
**HAL**	8.0	9.4	8.6	0.4	9.9	11.6	10.7	1.2
**FOL**	4.2	5.5	4.9	0.4	5.5	6.5	6.0	0.7

#### Etymology

The specific name is by the two Greek words *nekro-* (death) + -*nastes* (inhabitant; dweller). The name is given in allusion to the collection site of the specimens in a pond near a cemetery.

#### Advertisement call

**Fig 5:**
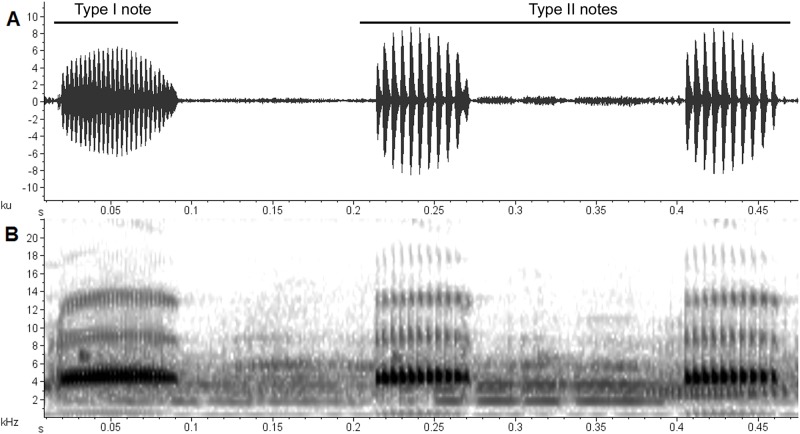
Advertisement call of *Dendropsophus nekronastes* sp. nov. (MZUESC 10175). (A) Waveform and (B) audiospectrogram. The first note is an example of a "Type I" note, while the second and third are "Type II" notes. Recorded on 02 February 2012 at 21 h 30 min. Air temperature during recording = 18.2°C.

The advertisement call of *Dendropsophus nekronastes* **sp. nov**. is composed by one (27%), two (41%) or three (32%) multipulsed notes (Fig 5). Call duration ranges between 0.052–0.501 s (0.305 ± 0.13) with 11–51 (35.5 ± 10.4) pulses per call. Pulse repetition rate is of 131.1 ± 29.7 pulses/s (90.5–211.5). Call rate is of 8.7 calls/min. Dominant frequency is also the fundamental frequency and ranges between 4478.9–4823.4 Hz (4598.9 ± 103). Two secondary harmonics bands can be discerned having a frequency between 8780–9150 Hz and 11385–13900 Hz, respectively. The first note has different duration, number of pulses and pulse repetition rate than the other two notes (Fig 5 and Table 2); the first can be interpreted as a “Type I” note, while the second and third as “Type II” notes due to their similitude. The "Type I" note may have a discrete ascending frequency modulation in its initial region, followed by a discrete descending modulation toward its final portion. The "Type II" note has a slight descending modulation in final region. The dominant frequency is similar between the two note types. In eight of the 56 analyzed calls, the pulses of the first note where not well individualized; in these cases, the related variables where not measured. Numeric data is summarized in Table 2.

**Table 2:** Means ± standard deviation and ranges (in parentheses) of acoustic parameters of advertisement call of *Dendropsophus leucophyllatus* Group. n = number of individuals; I = primary/introductory or Type I and II = number of secondary or Type II notes.

Species and number of recorded males	N° of notes*	Call Duration (s)	pulses per call	Note Duration (s)–I	Note Duration (s)–II	Pulses/note—I	Pulses/note—II	Pulse rate—I	Pulse rate—II	Call pulse repetition rate	Calls/min.	Dominant frequency (Hz)	Reference
***D*. *anceps* (n = 6)**	1 + 2–10	0.95 (0.44–1.4)	–	0.120 ± 0.022 (0.093–0.183)	0.043 ± 0.0053 (0.035–0.057)	12 ± 2 (10–18)	6 ± 1.2 (4–8)	–	–	–	–	3244 ± 114 (2997–3435)	Gomes and Martins [36]
***D*. *anceps* (n = 2)**	1 + ~ 6	1.06 ± 0.32 (0.74–1.88)	–	0.16 ± 0.04 (0.12–0.29)	0.07 ± 0.007 (0.06–0.09)	11.82 ± 1.78 (8–15)	5.4 ± 0.85 (4–7)	–	–	–	–	1.526 ± 24.9 (1.453.1–1565.4)	Conte et al. [37]
***D*. *bifurcus (n =*?*)***	1 + 0–2	0.197 ± 0.051 (0.092–0.305)	–	0.09 ± 0.009 (0.078–0.101)	0.020 ± 0.008.4 (0.010–0.033)	7.8 ± 0.79 (7–9)	2 ± 0.67 (1–3)	86.7 ± 3.5 (84.2–90.1)	101.4 ± 14.4 (76.9–111.1)	–	–	2960 ± 30 (2890–2980)	Jungfer et al. [17]
***D*. *bifurcus* (n = 8)**	1 + 1–3	–	–	0.09 (0.05–0.13)	–	–	–	–	–	102 (100–120)	23.1(15.3–35.3)	2971(2684–3339)	Duellman and Pyles [38]
*D*. ebraccatus** **(n = 43)**	1 + 2–5	–	–	0.19 (0.12–0.29)	0.03 (0.02–0.05)	14.2 (11–17)	–	93.3 (88–102)	–	–	–	2732 (2300–3450)	Duellman [19]
***D*. *ebraccatus* (n = 25)**	1 + 0–4	–	–	0.16 (0.12–0.20)	–	–	–	95 (90–100)		–	–	2500–3500	Wells and Greer [28]
***D*. *ebraccatus* (n = 6)**	1 + 2–5	0.20 (0.16–0.23)	–	–	–	–	–	–	–	92 (88–95)	–	2577 (2520–2620)	Duellman and Pyles [38]
***D*. *elegans* (n = 5)**	1 + 0	0.14 ± 0.04 (0.09–0.21)	14.9 ± 3.1 (9–22)	–	–	–	–	–	–	–	–	2000–3000	Bastos and Haddad [39]
***D*. *elegans* (n = 7)**	2	0.08 ± 0.013	–	0.05 ± 0.02	0.01 ± 0.003	8.7 ± 2.8 (5–16)	2.6 (2–5)	–	–	–	–	3570 ± 284 (2720–3910)	Muniz et al. [40]
***D*. *leucophyllatus (n =*?*)***	6 a 18 + 0	0.175 ± 0.025 (0.145–0.221)	15.7 ± 2.1 (13–19)	–	–	–	–	–	–	89.9 ± 5 (79–100)	143 ± 40.5 (4.3–178)	2481.8 ± 34.1 (2412–2562)	Marquez et al. [41]
***D*. *leucophyllatus* (n = 7)**	1–5 + 1–2	0.2 (0.13–0.27)	–	–	–	–	–	–	–	110 (100–130)	65.7 (17.1–120)	2283 (2056–2522)	Duellman and Pyles [38]
***D*. *leucophyllatus* (n = 4)**	1–3 + 0–5	0.13 (0.09–0.19)	–	–	–	–	–	–	–	192 (167–240)	17.9 (9–34.3)	2807 (2056–2522)	Duellman and Pyles [38])
***D*. *nekronastes* (n = 2)**	**1 + 0–2**	**0.305 ± 0.13 (0.052–0.501)**	**35.5 ± 10.4 (11–51)**	**0.106 ± 0.047 (0.052–0.201)**	**0.057 ± 0.004 (0.05–0.07)**	**25.6 ± 5 (11–32)**	**10.13 ± 0.68 (8–12)**	**265 ± 101.2 (144.1–391.3)**	**176.1 ± 12.19 (148.1–211.5)**	**131.1 ± 29.7(90.5–211.5)**	**8.7**	**4598.9 ± 103.5 (4478.9–4823.4)**	**this study**
***D*. *salli* (n =?)**	1 + 2–3	0.261 ± 0.026 (0.212–0.282)	–	0.071 ± 0.008 (0.061–0.080)	0.010 ± 0.002 (0.005–0.015)	20 ± 2 (16–23)	1.89 ± 0.4 (1–3)	281 ± 18.8 (259–391)	182 ± 38.1 (111–222)	–	–	2890 ± 70 (2850–3000)	Jungfer et al. [17]
*D*. salli*** **(n =?)**	1 + 1–3	0.147 ± 0.057 (0.092–0.220)	15.8 ± 1.5 (14–18)	–	–	–	–	–	–	138.6 ± 13.1 (123–158)	118.8 ± 114.6 (26–371)	2957.6 ± 153.1 (2692–3230)	Marquez et al. [41]
***D*. *sarayacuensis* (n = 8)**	1 + 1	0.08 (0.03–0.11)	–	–	–	–	–	–	–	78 (60–80)	26.8 (22.2–33.3)	2990 (2848–3326)	Duellman and Pyles [38]
***D*. *triangulum* (n = 4)**	1 + 3–5	0.17 (0.09–0.25)	–	–	–	–	–	–	–	180	21 (12–31.6)	2298 (2054–2535)	Duellman and Pyles [38]

* = number of primary/introductory or Type I notes + number of secondary or Type II notes.

** = Duellman [19] reports different values in the text and table. We reproduce the largest range and mean of the reported values.

*** = Advertisement call originally described as of *D*. *bifurcus*. Jungfer et al. [17] attributed this call to *D*. *salli*.

#### Phylogenetic relationships

**Fig 6:**
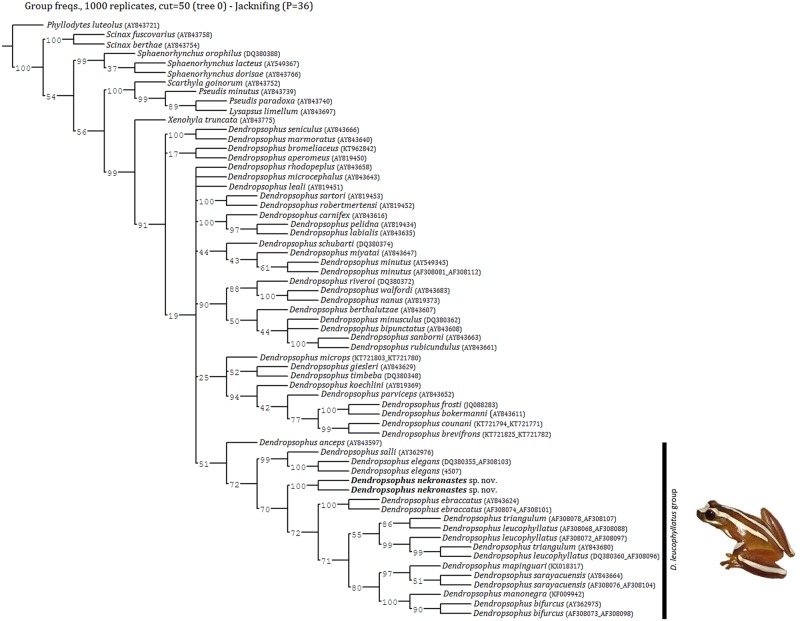
Phylogenetic relationship of *Dendropsophus nekronastes* sp. nov inferred by Maximum Parsimony. Strict consensus of the 18 most parsimonious trees (7,828 steps) obtained for the maximum parsimony analysis depicting the phylogenetic relationships among analyzed species (see Methods for analysis details). Numbers at nodes are Jackkinfe (JK) values based on 1000 pseudoreplicates.

Maximum Parsimony searches retrieved 18 equally most parsimonious trees with 7,828 steps. The consensus tree is shown in Fig 6. Our results recover *Dendropsophus* as the monophyletic sister taxon of *Xenohyla truncata*. The monophyly of the *D*. *leucophyllatus* Group (sensu [10]) is corroborated by our dataset. Regarding the new taxon, our phylogenetic analyses firmly places it within the *D*. *leucophyllatus* Group as sister species to a clade composed of *D*. *ebraccatus* + Amazonian species of the group (Fig 6). The mitochondrial sequence (16S rRNA gene) divergence (i.e. uncorrected pairwise distances) between *D*. *nekronastes* and the remaining species of the Group varies between 11.7–14.5% and is presented in Table 3. Conflict is widespread through the tree and, of the testable groups, only the *D*. *labialis*, *D*. *leucophyllatus* and the *D*. *marmoratus* Groups are monophyletic—a result similar to previous studies using similar databases and not relevant to our discussion given the position of our new taxon. We also retrieve *D*. *leucophyllatus* and *D*. *triangulum* as paraphyletic in respect to each other but this may be due to a number of issues and lays outside the scope of the present contribution (see [11]). Supports for more basal relationships are low (as in previous contributions).

**Table 3:** Genetic divergence (%) among species of the *Dendropsophus leucophyllatus* Group. Values are estimated from a dataset constructed from 808 bp of the 16S rRNA gene.

	Species	GenBank	1	2	3	4	5	6	7	8	9	10	11	12	13	14	15
**1**	*D*. *anceps*	AY843597	-														
**2**	*D*. *bifurcus*	AY362975	13.5	-													
**3**	*D*. *ebraccatus*	AY843624	13.0	13.0	-												
**4**	*D*. *elegans*	AF308103	12.7	13.3	13.2	-											
**5**	*D*. *elegans*	KY552469	13.5	13.0	12.0	4.1	-										
**6**	*D*. *leucophyllatus*	AF308096	13.1	11.3	11.9	13.6	13.5	-									
**7**	*D*. *leucophyllatus*	AF308088	11.3	11.5	12.3	13.7	13.7	9.2	-								
**8**	*D*. *manonegra*	KF009943	13.6	1.8	12.3	13.4	13.1	11.1	11.0	-							
**9**	*D*. *mapinguari*	KX018317	13.7	12.9	13.5	14.6	14.1	10.9	12.2	12.3	-						
**10**	*D*. *nekronastes*	KY552471	11.9	12.9	12.8	13.7	13.0	13.3	11.8	12.4	14.5	-					
**11**	*D*. *nekronastes*	KY552470	11.7	12.8	12.7	13.7	13.0	13.2	11.7	12.3	14.4	0.3	-				
**12**	*D*. *salli*	AY362976	14.3	12.5	14.6	11.2	10.7	13.7	14.5	13.3	15.7	14.2	14.2	-			
**13**	*D*. *sarayacuensis*	AY843664	13.2	10.4	12.9	14.1	13.8	11.3	11.1	10.5	10.4	13.3	13.2	13.9	-		
**14**	*D*. *triangulum*	AY843680	13.0	11.1	11.3	13.7	13.6	0.8	9.0	11.0	10.8	13.3	13.2	13.9	11.3	-	
**15**	*D*. *triangulum*	AF308107	12.3	11.8	11.8	13.3	13.0	8.5	6.8	10.9	12.1	11.1	10.8	13.9	11.6	8.4	-

**Fig 7:**
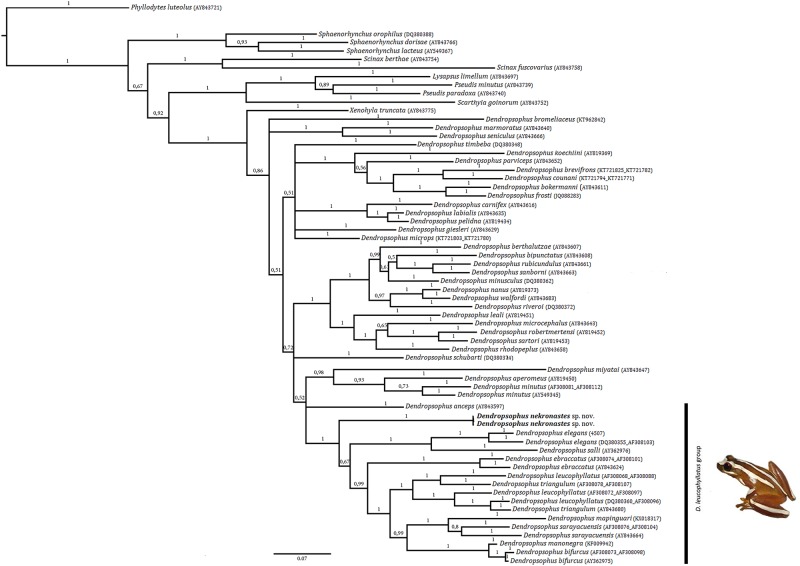
Topology inferred from Bayesian analysis. Numbers adjacent to nodes indicate posterior probabilities (see Methods for analysis details).

Bayesian Inference retrieves a similar result (Fig 7) and although slightly better resolved. The *D*. *labialis*, *D*. *marmoratus* and the *D*. *minutus* Groups are monophyletic while the remainders are not. Overall posterior probabilities are low (i.e. under 0.9) for most basal relationships within Dendropsophus. Most species of the *D*. *leucophyllatus* Group are recovered as a clade that is nested in a poorly supported polytomy (0,52) with *D*. *anceps* and a clade composed of *D*. *myiatai* + the *D*. *minutus* Group.

#### Comparison with other species of the *D*. *leucophyllatus* Group

*Dendropsophus nekronastes* differs from all other species of the Group by its unique dorsal pattern with a regular white frame resembling an inverted U-shape and a long white medial line (the sacral stripe) that starts posteriorly at the sacrum and extends anteriorly to the head (absent in all other species of the group); presence of two nearly transversal white bars on forelimbs (absent in all other species of the group); mucronate snout in dorsal view (truncate to rounded in all other species); advertisement call with the highest dominant frequency for the group—4478.0–4823.4 Hz (ranging 1453.1–3910 Hz in other species, see Table 2). Furthermore, *D*. *nekronastes* differs from all other species of the group (except *D*. *ebraccatus*) by the presence of a wide white suborbital stripe (when present, small, not as long as orbit diameter).

*Dendropsophus nekronastes* males are similar in size to most species of the group, with the exception of being larger than *D*. *rossalleni* and smaller than *D*. *anceps* and *D*. *leucophyllatus*. Females follow the same pattern but are also smaller than *D*. *triangulum* females. See Table 4 for values.

**Table 4:** Sizes of species in the *Dendropsophus leucophyllatus* Group.

SVL	*Males*		*Females*	
	Min	Max	Min	Max
*D*. *anceps*	31 ^a^	40 ^a^	39 ^a^	42 ^a^
*D*. *bifurcus*	23 ^b^	28 ^b^^,^^c^	29 ^b^	35 ^c^
*D*. *ebraccatus*	23.1 ^d^	29.3 ^d^	30 ^e^	36.8 ^f^
*D*. *elegans*	20 ^a^	29.6 ^g^	28.8 ^g^	36 ^a^
*D*. *leucophyllatus*	30.4 ^h^	36 ^b^	37.7 ^h^	50 ^i^
*D*. *mapinguari*	22.6 ^m^	24.8 ^m^	-	-
***D*. *nekronastes***	**24.1** ^n^	**28.9** ^n^	**31.8** ^n^	**35.4** ^n^
*D*. *rossalleni*	18 ^b^	22.3 ^j^	22 ^j^	28.7 ^j^
*D*. *salli*	25.4*^k^	30.1 ^l^	32 ^l^	32.5 **^l^
*D*. *sarayacuensis*	24 ^b^	29 ^b^	34 ^b^	37 ^b^
*D*. *triangulum*	24 ^b^	28 ^b^	36 ^b^	42 ^b^

^a^ = Lutz [42];

^b^ = Rodríguez and Duellman [43];

^c^ = Duellman [44];

^d^ = Duellman, [19];

^e^ = Savage [45];

^f^ = Cochran and Goin [18];

^g^ = Bastos and Haddad [46];

^h^ = Caldwell and Araújo [47];

^i^ = Lescure and Marty [48];

^j^ = De la Riva and Duellman [49];

^k^ = Marquez et al. [41];

^l^ = Jungfer et al. [17];

^m^ = Peloso et al. [11];

^n^ = present paper;

* = X-SD provided in the paper;

** = X+SD provided in the paper

#### Geographic distribution

*Dendropsophus nekronastes* **sp. nov.** is known only from two ponds at its type-locality, municipality of Almadina, southern State of Bahia, Brazil (Fig 1).

#### Natural history

*Dendropsophus nekronastes* call in temporary ponds, usually in the surrounding shrubs between 30 and 150 cm of height. Syntopical congeners are *D*. *branneri*, *D*. *elegans* (MZUESC 10186–10194) and *D*. *anceps* (MZUESC 9960–9964). Other species calling at the sites were *Hypsiboas albomarginatu*s, *H*. *crepitans*, *H*. *faber*, *Phyllomedusa burmeisteri*, *P*. *rohdei*, *Physalaemus erikae*, *Scinax juncae*, *Sphaenorhynchus pauloalvini* and *S*. *prasinus*.

## Discussion

*Dendropsophus nekronastes* was found in ponds located at anthropogenically-disturbed areas; like the city cemetery. The surroundings are cacao plantations and open pasturelands. The region (Almadina and surrounding municipalities) does not have any protected areas. Although known only from its type locality, *D*. *nekronastes* does not seem to require a pristine habitat. Many *Dendropsophus* species are widespread [50–53] and most of those are commonly found on slightly disturbed areas (see [54])—apparently, *D*. *nekronastes* fits this pattern. Nevertheless, our lack of knowledge about its ecological requirements precludes any evaluation about the species tolerance to anthropization or if it occurs in this—restricted—disturbed area due to the lack of more pristine habitats. Another option is that *D*. *nekronastes* is a canopy dweller (see below).

For the Bahia southern sector, only *D*. *nekronastes* and 10 other anuran species are known only from their type-localities [8,55–63]. Most seem explosive breeders (sensu [64]) and/or canopy dwellers, which are less likely to be collected [65]; if *D*. *nekronastes* present such life patterns is unknown. Nevertheless, the last years witnessed many amphibian surveys in the region [2,3,66], including one that sampled Almadina [67], and because *Dendropsophus* species usually breed in ponds (e.g., [46]), an environment that herpetologists usually highly prioritize as collecting sites due to their accessibility, it is remarkable that *D*. *nekronastes* has not been found before. *Dendropsophus* is highly speciose and new species have been found in well-collected sites before (e.g., [9]). Within nine field trips to Almadina, *D*. *nekronastes* was found only in four in different densities. In the first two, when the type series was collected, there were many individuals calling. In the latter two, only a few individuals were calling (and none was collected). Therefore it is possible that *D*. *nekronastes* is an explosive breeders and/or canopy dweller with larger geographic distribution. However, given that it is only known from a single population, it becomes susceptible to urban expansion and farming.

Most species of the *D*. *leucophyllatus* Group have known calls—exceptions are *D*. *mapinguari*, *D*. *manonegra* and *D*. *rossalleni*—but measured parameters (even for the same species in different contributions) vary among different authors. The only comparable variables are: number and type of notes, call or note duration, and dominant frequency. All species of the *D*. *leucophyllatus* Group have biphasic calls [17,37,40,41] with one note primary (also called “main”, “introductory” or, “Type I”) that is usually longer and with more pulses and is followed by one to ten shorter secondary (or “Type II”) notes [28,41].

Secondary notes are not always produced but it is known that in amphibians these are more frequent in chorus situation [28,41]. Nevertheless, it has been demonstrated that some populations of *D*. *elegans* never produce them [39]; other *Dendropsophus* species are also known to have plastic calls and isolated and chorusing males produce different calls [68,69].

*Dendropsophus* internal topology is still unstable [11]. Different optimality criteria and alignment methods result in radically different topologies but most internal nodes have low support—exactly what we have also found. There are a series of reasons why this may happens and for a specific discussion on *Dendropsophus* topologies, see Peloso et al. [11].

Nevertheless, the presence of a biphasic call may be a synapomorphy for some groups or internal clades of the genus. *Dendropsophus nekronastes* and *D*. *elegans* are somewhat similar—although their calls are quite different. In fact, the distinct advertisement calls of *D*. *nekronastes* and *D*. *elegans* were the first clue that they would be different species. Overall, although similar to the calls of other species of the Group, *D*. *nekronastes* call has a higher dominant frequency.

Our results suggest an interesting biogeographic history of the species in the *D*. *leucophyllatus* Group. For the moment it is impossible to undoubtedly understand their distributions in a historical perspective—especially given the highly unstable phylogeny of the genus. However, *D*. *salli* is an Amazon dweller and may represent an independent colonization of this biome when compared to the other species of the group.

Notwithstanding, both MP and BI analyses imply that *D*. *nekronastes* is not directly related (i.e. sister species) to *D*. *anceps* and *D*. *elegans* that are syntopic with the new species. This scenario may be another example of the past relationship between Northern Espírito Santo–Southern Bahia (Hiléia Bahiana) and the Amazon. Within MP this relationship is marginally supported (71% JK; see Fig 6) however. This past connection has been suggested before [6–8,70–72] and two possible connections have been hypothesized [7,51,73]. Additional studies with other taxa may confirm and date such connections.

MP recovers *D*. *anceps* as sister species of the remainder species of the *D*. *leucophyllatus* Group with low support while BI does not provide an indubitable solution for this matter. As previously reported, the allocation of *D*. *anceps* is a matter of ongoing discussion [11]. It seems clear that this issue ill be only adequately dealt within a comprehensive analysis of *Dendropsophus*.

## Supporting information

S1 Appendix Additional specimens examined. (DOCX)

S2 Appendix GenBank accession numbers for hylid frog sequences (12S, 16S rRNA and valine-tRNA) used for this study. (DOCX)

S3 Appendix Measurements morphometric of each individual analyzed of the type-series of *Dendropsophus nekronastes* sp. nov. (DOC)

S1 File Input file used in TNT for Maximum Parsimony analysis. (TNT)

S2 File Input file used in MrBayes 3.2.6 for Bayesian analysis. (NEX)

## References

[1] Izecksohn E, Peixoto OL (1981) Nova espécie de *Proceratophrys*, da Hiléia Bahiana, Brasil (Amphibia, Anura, Leptodactylidae). Revista Brasileira de Biologia 41: 19–24.

[2] Silvano DL, Pimenta BVS (2003) Diversidade e distribuição de anfíbios anuros na Mata Atlântica do sul da Bahia In: Prado PI, Landau EC, Moura RTd, Pinto LPS, Fonseca GABd et al, editors. Corredor de Biodiversidade da Mata Atlântica do Sul da Bahia. Ilhéus: IESB/CI/CABS/UFMG/UNICAMP. pp. CD-ROM.

[3] Dias IR, Medeiros T, Vila Nova M, Solé M (2014) Amphibians of Serra Bonita, southern Bahia: a new hotpoint within Brazil’s Atlantic Forest hotspot. ZooKeys 449: 105–130. 10.3897/zookeys.449.7494 PMC4233400 25408616

[4] Napoli MF, Cruz CAGd, Abreu ROd, Del-Grande ML (2011) A new species of *Proceratophrys* Miranda-Ribeiro (Amphibia: Anura: Cycloramphidae) from the Chapada Diamantina, State of Bahia, northeastern Brazil. Zootaxa 3133: 37–49.

[5] Teixeira M Jr, Amaro RC, Recoder RS, Dal Vechio F, Rodrigues MT (2012) A new dwarf species of *Proceratophrys* Miranda-Ribeiro, 1920 (Anura, Cycloramphidae) from the highlands of Chapada Diamantina, Bahia, Brazil. Zootaxa 3551: 25–42.

[6] Costa LP (2003) The historical bridge between the Amazon and the Atlantic Forest of Brazil: a study of molecular phylogeography with small mammals. Journal of Biogeography 30: 71–86.

[7] Fouquet A, Loebmann D, Castroviejo-Fisher S, Padial JM, Orrico VGD, Lyra ML, et al (2012) From Amazonia to the Atlantic forest: Molecular phylogeny of Phyzelaphryninae frogs reveals unexpected diversity and a striking biogeographic pattern emphasizing conservation challenges. Molecular Phylogenetics and Evolution 65: 547–561. 10.1016/j.ympev.2012.07.012 22842094

[8] Caramaschi U, Orrico VGD, Faivovich J, Dias IR, Solé M (2013) A New Species of Allophryne (Anura: Allophrynidae) from the Atlantic Rain Forest Biome of Eastern Brazil. Herpetologica 69: 480–491.

[9] Motta AP, Castroviejo-Fisher S, Venegas PJ, Orrico VGD, Padial JM (2012) A new species of the *Dendropsophus parviceps* group from the western Amazon Basin (Amphibia: Anura: Hylidae). Zootaxa 3249: 18–30.

[10] Rivera-Correa M, Orrico VGD (2013) Description and phylogenetic relationships of a new species of treefrog of the *Dendropsophus leucophyllatus* group (Anura: Hylidae) from the Amazon basin of Colombia and with an exceptional color pattern. Zootaxa 3686: 447–460. 26473232 10.11646/zootaxa.3686.4.3

[11] Peloso PLV, Orrico VGD, Haddad CFB, Lima-Filho GR, Sturaro MJ (2016) A New Species of Clown Tree Frog, *Dendropsophus leucophyllatus* Species Group, from Amazonia (Anura, Hylidae). South American Journal of Herpetology 11: 66–80.

[12] Orrico VGD, Peloso PLV, Sturaro MJ, Silva-Filho HF, Neckel-Oliveira S, Gordo M, et al (2014) A new “Bat-Voiced” species of *Dendropsophus* Fitzinger, 1843 (Anura, Hylidae) from the Amazon Basin, Brazil. Zootaxa 3881: 341–361. 10.11646/zootaxa.3881.4.3 25543640

[13] Carvalho-e-Silva SPd, Carvalho-e-Silva AMPTd, Izecksohn E (2003) Nova espécie de *Hyla* Laurenti do grupo de *H*. *microcephala* Cope (Amphibia, Anura, Hylidae) do nordeste do Brasil. Revista Brasileira de Zoologia 20: 553–558.

[14] Ferreira RB, Faivovich J, Beard KH, Pombal JP Jr. (2015) The First Bromeligenous Species of Dendropsophus (Anura: Hylidae) from Brazil's Atlantic Forest. PLoS ONE 10: e0142893 10.1371/journal.pone.0142893 26650515 PMC4674083

[15] Frost D (2016) Amphibian Species of the World: an Online Reference. Version 6.0. New York: American Museum of Natural History.

[16] Faivovich J, Haddad CFB, Garcia PCA, Frost DR, Campbell JA, Wheeler WC, et al (2005) Systematic review of the frog family Hylidae, with special reference to Hylinae: phylogenetic analysis and taxonomic revision. Bulletin of the American Museum of Natural History 294: 1–240.

[17] Jungfer K-H, Reichle S, Piskurek O (2010) Description of a new cryptic southwestern Amazonian species of leaf-gluing treefrog, genus *Dendropsophus* (Amphibia: Anura: Hylidae). Salamandra 46: 204–213.

[18] Cochran DM, Goin CJ (1970) Frogs of Colombia. Bulletin of the United States National Museum 288: 1–655.

[19] Duellman WE (1970) Hylid frogs of Middle America Monographs of the Museum of Natural History, University of Kansas 1–2: 1–753.

[20] Duellman WE (1966) Taxonomic notes on some Mexican and Central American Hylid frogs. University of Kansas Publications, Museum of Natural History 17: 263–279.

[21] Duellman WE (2001) Hylid frogs of Middle America. Volume 1 i–xvi, 1–694 p.

[22] Napoli MF, Caramaschi U (1998) Duas novas especies de *Hyla* Laurenti, 1768 do Brasil central afins de *H*. *tritaeniata* Bokermann, 1965 (Amphibia, Anura, Hylidae). Boletim do Museu Nacional—Nova Série, Zoologia 391: 1–12.

[23] Savage JM, Heyer WR (1967) Variation and distribution in the tree-frog genus *Phyllomedusa* in Costa Rica, central America. Beitrage Zur Neotropischen Fauna 5: 111–131.

[24] Savage JM, Heyer WR (1997) Digital webbing formulae for amphibians: a refinement. Herpetological Review 28: 131.

[25] Myers CW, Duellman WE (1982) A new species of *Hyla* from Cerro Colorado, and other tree frog records and geographical notes from western Panama. American Museum Novitates: 1–32.

[26] Duellman WE, Trueb L (1986) Biology of Amphibians. New York: McGraw-Hill.

[27] Heyer WR, Rand AS, Cruz CAG, Peixoto OL, Nelson CE (1990) Frogs of Boracéia. Arquivos de Zoologia 31: 231–410.

[28] Wells KD, Greer BJ (1981) Vocal Responses to Conspecific Calls in a Neotropical Hylid Frog, *Hyla ebraccata*. Copeia 1981: 615–624.

[29] Duellman WE (1967) Courtship isolating mechanisms in Costa Rican hylid frogs. Herpetologica 23: 169–183.

[30] Katoh K, Misawa K, Kuma K-i, Miyata T (2005) MAFFT- a novel method for rapid multiple sequence alignment based on fast Fourier transform. Nucleic Acids Research 30: 3059–3066. 10.1093/nar/gkf436 PMC135756 12136088

[31] Goloboff PA, Farris JS, Nixon KC (2008) TNT, a free program for phylogenetic analysis. Cladistics 24: 774–786.

[32] Ronquist F, Teslenko M, Mark Pvd, Ayres D, Darling A, Höhna S, et al (2012) MrBayes 3.2: Efficient Bayesian phylogenetic inference and model choice across a large model space. Systematic Biology 61: 539–542. 10.1093/sysbio/sys029 22357727 PMC3329765

[33] Miller MA, Pfeiffer W, Schwartz T (2010) Creating the CIPRES Science Gateway for inference of large phylogenetic trees. Proceedings of the Gateway Computing Environments Workshop (GCE). New Orleans. pp. 1–8.

[34] Lanfear R, Calcott B, Ho SY, Guindon S (2012) Partitionfinder: combined selection of partitioning schemes and substitution models for phylogenetic analyses. Molecular Biology and Evolution 29: 1695–1701. 10.1093/molbev/mss020 22319168

[35] Rambaut A, Suchard MA, Xie D, Drummond AJ (2014) Tracer v1.6. http://beastbioedacuk/Tracer.

[36] Gomes FBR, Martins IA (2006) Notes on Geographic Distribution. Amphibia, Anura, Hylidae, *Dendropsophus anceps* (Lutz, 1929): filling gap, geographic distribution map and vocalization. Check List 2: 22–25.

[37] Conte CE, Nomura F, Machado RA, Kwet A, Lingnau R, Rossa-Feres DC (2010) Novos registros na distribuição geográfica de anuros na Floresta com Araucária e considerações sobre suas vocalizações. Biota Neotropica 10: 201–224.

[38] Duellman WE, Pyles RA (1983) Acoustic Resource Partitioning in Anuran Communities. Copeia 1983: 639–649.

[39] Bastos RP, Haddad CFB (1995) Vocalizações e interações acústicas de *Hyla elegans* (Anura, Hylidae) durante a atividade reprodutiva Naturalia 20: 165–176.

[40] Muniz SLdS, Moura CCdM, Moraes ATdA, Galindo MKF, Chaves LdS, Kokubum MNC et al (2016) Acoustic characteristics of the advertisement call of *Dendropsophus elegans* (Anura: Hylidae). Herpetology Notes 9: 99–102

[41] Marquez R, De la Riva I, Bosch J (1993) Advertisement calls of bolivian species of *Hyla* (Amphibia, Anura, Hylidae). Biotropica 25: 426–443.

[42] Lutz B (1973) Brazilian species of *Hyla*. San Antonio, Texas, USA: University of Texas Press, Austin & London i-xviii, 1–265 p.

[43] Rodriguez LO, Duellman WE (1994) Guide to the frogs of the Iquitos region, Amazonian Perú; Trueb L, editor. Lawrence, Kansas: Asociación de Ecologia y Conservación, Amazon Center for Environmental Education and Research, and Natural History Museum, The University of Kansas.

[44] Duellman WE (1978) The biology of an equatorial herpetofauna in Amazonian Ecuador. Miscellaneous Publications of the Museum of Natural History of the University of Kansas 65: 1–352.

[45] Savage JM (2002) The amphibians and reptiles of Costa Rica: a herpetofauna between two continents, between two seas. The amphibians and reptiles of Costa Rica: a herpetofauna between two continents, between two seas: University of Chicago Press pp. i–xx, 1–934.

[46] Bastos RP, Haddad CFB (1996) Breeding Activity of the Neotropical Treefrog *Hyla elegans* (Anura, Hylidae). Journal of Herpetology 30: 355–360.

[47] Caldwell JP, Araújo MCd (2005) Amphibian Faunas of Two Eastern Amazonian Rainforest sites in Pará, Brazil. Occasional Papers Sam Noble Oklahoma Museum of Natural History 16: 1–41.

[48] Lescure J, Marty C (2000) Atlas des Amphibiens de Guyane. Patrimoines Naturels 45: 1–388.

[49] de la Riva I, Duellman WE (1997) The identity and distribution of *Hyla rossalleni* Goin. Amphibia-Reptilia 18: 433–436.

[50] Fouquet A, Noonan BP, Blanc M, Orrico VGD (2011) Phylogenetic position of *Dendropsophus gaucheri* (Lescure and Marty 2000) highlights the need for an in-depth investigation of the phylogenetic relationships of *Dendropsophus* (Anura: Hylidae). Zootaxa 3035: 59–67.

[51] Gehara M, Crawford AJ, Orrico VGD, Rodríguez A, Lötters S, Fouquet A, et al (2014) High levels of diversity uncovered in a widespread nominal taxon: continental phylogeography of the Neotropical tree frog *Dendropsophus minutus*. PLoS ONE 9: e103958 10.1371/journal.pone.0103958 25208078 PMC4160190

[52] Zina J, Ramos da Silva G, Loebmann D, Orrico VGD ("2014"[2015]) The recognition of *Dendropsophus minusculus* (Rivero, 1971) (Hylidae, Dendropsophini) as a highly polymorphic, multi-domain distributed species. Brazilian Journal of Biology 74: S146–S153. 10.1590/1519-6984.22912 25627378

[53] Orrico VGD, Duellman WE, Souza MB, Haddad CFB (2013) The Taxonomic Status of *Dendropsophus allenorum* and *D*. *timbeba* (Anura: Hylidae). Journal of Herpetology 46: 615–618.

[54] Izecksohn E, Carvalho-e-Silva SPd (2001) Anfíbios do Município do Rio de Janeiro. Rio de Janeiro: Editora UFRJ.

[55] Napoli MF, Caramaschi U, Cruz CAGd, Dias IR (2011) A new species of flea-toad, genus *Brachycephalus* Fitzinger (Amphibia: Anura: Brachycephalidae), from the Atlantic rainforest of southern Bahia, Brazil. Zootaxa 2739: 33–40.

[56] Canedo C, Dixo M, Pombal JP Jr. (2004) A new species of *Chiasmocleis* Méhelÿ, 1904 (Anura, Microhylidae) from the atlantic rainforest of Bahia, Brazil. Herpetologica 60: 495–501.

[57] Teixeira M Jr, Recoder RS, Amaro RC, Damasceno RP, Cassimiro J, Rodrigues MT, et al (2013) A new *Crossodactylodes* Cochran, 1938 (Anura: Leptodactylidae: Paratelmatobiinae) from the highlands of the Atlantic Forests of southern Bahia, Brazil. Zootaxa 3702: 459–472. 26146739 10.11646/zootaxa.3702.5.5

[58] Juncá FA, Nunes I (2008) A new species of marsupial frog of the genus *Gastrotheca* Fitzinger (Anura: Amphignatodontidae) from the State of Bahia, Northeastern Brazil. Zootaxa 1907: 61–68.

[59] Caramaschi U (2012) A new species of beaked toad, *Rhinella* (Anura: Bufonidae), from the State of Bahia, Brazil. Zoologia 29: 343–348.

[60] Bokermann WCA (1966) Duas novas especies de Sphaenorhynchus (Amphibia, Hylidae). Rev Brasil Biol 26: 15–21.

[61] Cruz CAGd, Caramaschi U, Napoli MF (2007) A new species of *Chiasmocleis* (Anura, Microhylidae) from the Atlantic Rain Forest of Northeastern Bahia, Brazil. South American Journal of Herpetology 2: 47–52.

[62] Carcerelli LC, Caramaschi U ("1992" [1993]) Ocorrência do gênero *Crossodactylus* Duméril & Bibron, 1841 no nordeste brasileiro, com descrição de duas espécies novas (Amphibia, Anura, Leptodactylidae). Revista Brasileira de Biologia 52: 415–422.

[63] Recoder R, Teixeira M Jr, Cassimiro J, Camacho A, Rodrigues MT (2010) A new species of *Dendrophryniscus* (Amphibia, Anura, Bufonidae) from the Atlantic Rainforest of southern Bahia, Brazil. Zootaxa 2642: 36–44.

[64] Wells KD (2007) The Ecology and Behavior of Amphibians. Chicago and London: University Of Chicago Press.

[65] Batista A, Hertz A, Mebert K, Köhler G, Lotzkat S, Ponce M, et al (2014) Two new fringe-limbed frogs of the genus *Ecnomiohyla* (Anura: Hylidae) from Panama. Zootaxa 3826: 449–474. 10.11646/zootaxa.3826.3.2 24990059

[66] Camurugi F, Lima TM, Mercês EdA, Juncá FA (2010) Anurans of the Reserva Ecológica da Michelin, Municipality of Igrapiúna, State of Bahia, Brazil. Biota Neotropica 10: 305–312.

[67] Dias IR, Mira-Mendes CV, Solé M (2014) Rapid inventory of herpetofauna at the APA (Environmental Protection Area) of the Lagoa Encantada and Rio Almada, Southern Bahia, Brazil. Herpetology Notes 7: 627–637.

[68] Martins I, Jim J (2003) Bioacoustic analysis of advertisement call in *Hyla nana* and *Hyla sanborni* (Anura, Hylidae) in Botucatu, São Paulo, Brazil Brazilian Journal of Biology 63: 507–516. 10.1590/s1519-69842003000300017 14758710

[69] Morais AR, Batista VG, Gambale PG, Signorelli L, Bastos RP (2012) Acoustic communication in a Neotropical frog (*Dendropsophus minutus*): vocal repertoire, variability and individual discrimination. Herpetological Journal 22: 249–257.

[70] Canedo C, Haddad CFB (2012) Phylogenetic relationships within anuran clade Terrarana, with emphasis on the placement of Brazilian Atlantic rainforest frogs genus *Ischnocnema* (Anura: Brachycephalidae). Molecular Phylogenetics and Evolution 65: 610–620. 10.1016/j.ympev.2012.07.016 22842090

[71] De Sa RO, Streicher JW, Sekonyela R, Forlani MC, Loader SP, Greenbaum E, et al (2012) Molecular phylogeny of microhylid frogs (Anura: Microhylidae) with emphasis on relationships among New World genera. BMC Evolutionary Biology 12. 10.1186/1471-2148-12-241 PMC3561245 23228209

[72] Fernandes DS, Franco FL, Fernandes R (2004) Systematic revision of the genus *Lachesis* Daudin, 1803 (Serpentes, Viperidae). Herpetologica 60: 245–260.

[73] Fouquet A, Cassini CS, Haddad CFB, Pech N, Rodrigues MT (2014) Species delimitation, patterns of diversification and historical biogeography of the Neotropical frog genus *Adenomera* (Anura, Leptodactylidae). Journal of Biogeography 41: 855–870.

