# Supplementary material for: The 100th: An appealing new species of Dendropsophus (Amphibia: Anura: Hylidae) from northeastern Brazil
Source: PLoS One. 2017 Mar 8;12(3):e0171678. doi: 10.1371/journal.pone.0171678 (PMC5342187; doi:10.1371/journal.pone.0171678)
Supplement: S1 Appendix — (DOCX) [file pone.0171678.s001.docx]

**S1 Appendix.** Additional specimens examined.

*Dendropsophus* *anceps*: BRAZIL: BAHIA: Almadina: MZUESC 9960-9964; Espírito Santo: Linhares: CFBH 5795–5804; São Paulo: Caçapava: CFBH 13208–13210.

*Dendropsophus* *bifurcus*: ECUADOR: Morona: Santiago: Sevilla Don Bosco: MZUSP 55637; Napo: Santa Cecilia: MZUSP 116703–116706, 116695, 116696; Loreto: MZUSP 116720–116724; Orellana: Estación Científica Yasuní PUCE: QCAZ 24396–24406; Pastaza: Rio Villano: MZUSP 117915; Rio Solís: MZUSP 76478.

*Dendropsophus* *ebraccatus*: COLOMBIA: Antioquia: Maceo: Las Brisas: 500m. MHUA-A 2159, 2590-2591, 4063, 4807–4810; HONDURAS: Gracias a Dios: USNM 559102, 559105, 559107, 550109–550113, 550115, 550116.

*Dendropsophus* *elegans*: BRAZIL: Bahia: Almadina: MZUESC 10186-10194, Caravelas: MZUESC 9852-9853 (topotypes), Guandú: CFBH 27974; Espírito Santo: Linhares: CFBH 22663-22667.

*Dendropsophus* *leucophyllatus*: ECUADOR: Orellana: Río Napo: Chiroisla: Banco norte: QCAZ 44455–44458.

*Dendropsophus manonegra*: COLOMBIA: Caqueta: Florencia: MHUA-A 7336-7337, 7668.

*Dendropsophus mapinguari*: BRAZIL: Amazonas: Itacoatiara, Lindóia: MNRJ 56732–56735.

*Dendropsophus* *rossalleni*: BRAZIL: Amazonas: CFBH 4988, 4990; Pará: Oriximiná: MCNAM 8671, 8672.

*Dendropsophus* *salli*: BRAZIL: Acre: Tarauacá: MZUSP 116707–116719; Rondônia: Porto Velho: MZUSP 117916- 117917, 116697–11702.

*Dendropsophus* *sarayacuensis*: BRAZIL: Mato Grosso: Aripuanã: MZUSP 80632–80634; Rondônia: Porto Velho: MZUSP 146192, 148388; ECUADOR: Napo: Río Salado: QCAZ 36699-36700, 36801, 36802.

*Dendropsophus* *triangulum*: ECUADOR: Orellana: Río Napo: Santa Teresita: Nuevo Rocafuerte: Banco norte del Río Napo: QCAZ 44667–44672.
