# Supplementary material for: The 100th: An appealing new species of Dendropsophus (Amphibia: Anura: Hylidae) from northeastern Brazil
Source: PLoS One. 2017 Mar 8;12(3):e0171678. doi: 10.1371/journal.pone.0171678 (PMC5342187; doi:10.1371/journal.pone.0171678)
Supplement: S2 Appendix — (DOCX) [file pone.0171678.s002.docx]

**S2 Appendix.** GenBank accession numbers for hylid frog sequences (12S, 16S rRNA and valine-tRNA) used for this study.

| **Taxon** | **Locality** | **12S** | **16S** | **valine-tRNA** | **Source for Sequences** |
| --- | --- | --- | --- | --- | --- |
| *Dendropsophus anceps* | Brazil: Espírito Santo: Linhares | AY843597 | AY843597 | AY843597 | Faivovich et al. (2005) |
| *Dendropsophus aperomeus* | Peru: San Martin: Rioja: Venceremos | AY819450 | — | — | Wiens et al. (2005) |
| *Dendropsophus berthalutzae* | Brazil: Rio de Janeiro: Duque de Caxias | AY843607 | AY843607 | AY843607 | Faivovich et al. (2005) |
| *Dendropsophus bifurcus* | Ecuador | AY362975 | AY362975 | AY362975 | Jungfer et al. (2010) |
| *Dendropsophus bifurcus* | Ecuador: Jatun Sacha, Napo | AF308073 | AF308098 | — | Check et al. (2001) |
| *Dendropsophus bipunctatus* | Brazil: Bahia: Jussari: Serra do Teimoso | AY843608 | AY843608 | AY843608 | Faivovich et al. (2005) |
| *Dendropsophus bokermanni* | Peru: Huanuco, Rio Llullapichis, Panguana | AY843611 | AY843611 | AY843611 | Faivovich et al. (2005) |
| *Dendropsophus brevifrons* | Ecuador: Limoncocha | KT721825 | KT721782 | — | Fouquet et al. (2015) |
| *Dendropsophus bromeliaceus* | Brazil: Espírito Santo: Santa Teresa | KT962842 | KT962842 | KT962842 | Ferreira et al. (2015) |
| *Dendropsophus carnifex* | Ecuador: Pichincha: Tandayapa | AY843616 | AY843616 | AY843616 | Faivovich et al. (2005) |
| *Dendropsophus counani* | French Guiana: Montagne tortue grande | KT721794 | KT721771 | — | Fouquet et al. (2015) |
| *Dendropsophus ebraccatus* | Belize: Stann Creek District | AY843624 | AY843624 | AY843624 | Faivovich et al. (2005) |
| *Dendropsophus ebraccatus* | Panama: Canal Zone, Gamboa | AF308074 | AF308101 | — | Check et al. (2001) |
| *Dendropsophus elegans* | Brazil: Bahia: Almadina | KY552469 | KY552469 | KY552469 | **This study** |
| *Dendropsophus elegans^1^* | — | DQ380355 | AF308103 | — | Wiens et al. (2006) and  Check et al. (2001) |
| *Dendropsophus frosti* | Colombia: Departamento Amazonas | JQ088283 | JQ088283 | JQ088283 | Motta et al. (2012) |
| *Dendropsophus giesleri* | Brazil: São Paulo: Ubatuba: Picinguaba | AY843629 | AY843629 | AY843629 | Faivovich et al. (2005) |
| *Dendropsophus koechlini* | Peru: Madre de Dios: Cuzco Amazonica | AY819369 | — | — | Wiens et al. (2005) |
| *Dendropsophus labialis* | Colombia: Parque Natural Nacional Chingaza | AY843635 | AY843635 | AY843635 | Faivovich et al. (2005) |
| *Dendropsophus leali* | Peru: Madre de Dios: Cuzco Amazonica | AY819451 | — | — | Wiens et al. (2005) |
| *Dendropsophus leucophyllatus* | Brazil: Amapá: Serra do Navio | AF308068 | AF308088 | — | Check et al. (2001) |
| *Dendropsophus leucophyllatus* | Brazil: Acre: Rio Branco | AF308072 | AF308097 | — | Check et al. (2001) |
| *Dendropsophus leucophyllatus^1^* | — | DQ380360 | AF308096 | — | Wiens et al. 2006 and  Check et al. (2001) |
| *Dendropsophus manonegra* | Colombia: Departamento de Caqueta: Municipio de Florencia | KF009942 | KF009942 | KF009942 | Rivera-Correa & Orrico (2013) |
| *Dendropsophus mapinguari* | Brazil: Amazonas: Maues - Floresta Nacional de Pau Rosa | KX018317 | KX018317 | KX018317 | Peloso et al. (2016) |
| *Dendropsophus marmoratus* | Peru: Huanuco: Rio Llullapichis: Panguana | AY843640 | AY843640 | AY843640 | Faivovich et al. (2005) |
| *Dendropsophus microcephalus* | Honduras: Atlantida: Cordillera Nombre de Dios | AY843643 | AY843643 | AY843643 | Faivovich et al. (2005) |
| *Dendropsophus microps* | Brazil: São Paulo: Pinheirinho | KT721803 | KT721780 | — | Fouquet et al. (2015) |
| *Dendropsophus minusculus* | Trinidad and Tobago: Trinidad: St. Patrick | DQ380362 | — | — | Wiens et al. (2006) |
| *Dendropsophus minutus* | Argentina: Misiones, Guarani, San Vicente, Campo | — | AY549345 | AY549345 | Faivovich et al. (2005) |
| *Dendropsophus minutus* | Brazil: Pará: A-Ukre | AF308081 | AF308112 | — | Check et al. (2001) |
| *Dendropsophus miyatai* | Ecuador: Sucumbios | AY843647 | AY843647 | AY843647 | Faivovich et al. (2005) |
| *Dendropsophus nanus* | Brazil: São Paulo | AY819373 | — | — | Wiens et al. (2005) |
| *Dendropsophus nekronastes* | Brazil: Bahia: Almadina | KY552471 | KY552471 | KY552471 | **This study** |
| *Dendropsophus nekronastes* | Brazil: Bahia: Almadina | KY552470 | KY552470 | KY552470 | **This study** |
| *Dendropsophus parviceps* | Brazil: Acre: Centro Experimental da Universidade do Acre | AY843652 | AY843652 | AY843652 | Faivovich et al. (2005) |
| *Dendropsophus pelidna* | Venezuela: Tachira: Betania | AY819434 | — | — | Wiens et al. (2005) |
| *Dendropsophus rhodopeplus* | Peru: Loreto, Jenaro Herrera | AY843658 | AY843658 | AY843658 | Faivovich et al. (2005) |
| *Dendropsophus riveroi* | Ecuador: Sucumbios | DQ380372 | — | — | Wiens et al. 2006 |
| *Dendropsophus robertmertensi* | Mexico: Oaxaca: Zanatepec | AY819452 | — | — | Wiens et al. (2005) |
| *Dendropsophus rubicundulus* | Brazil: São Paulo, Buri | AY843661 | AY843661 | AY843661 | Faivovich et al. (2005) |
| *Dendropsophus salli* | Bolivia | AY362976 | AY362976 | AY362976 | Jungfer et al. (2010) |
| *Dendropsophus sanborni* | Argentina: Entre Rios: Departamento Islas del Ibicuy | AY843663 | AY843663 | AY843663 | Faivovich et al. (2005) |
| *Dendropsophus sarayacuensis* | Peru: Huanuco: Rio Llullapichis: Panguana | AY843664 | AY843664 | AY843664 | Faivovich et al. (2005) |
| *Dendropsophus sarayacuensis* | Brazil: Altamira, Amazonas | AF308076 | AF308104 | — | Check et al. (2001) |
| *Dendropsophus sartori* | Mexico: Guerrero | AY819453 | — | — | Wiens et al. (2005) |
| *Dendropsophus schubarti* | — | DQ380374 | — | — | Wiens et al. (2006) |
| *Dendropsophus seniculus* | Brazil: Rio de Janeiro: Angra dos Reis | AY843666 | AY843666 | AY843666 | Faivovich et al. (2005) |
| *Dendropsophus timbeba* | Peru: Madre de Dios: Cuzco Amazonico | DQ380348 | — | — | Wiens et al. (2006) |
| *Dendropsophus triangulum* | Brazil: Amazonas: Tabatinga | AF308078 | AF308107 | — | Check et al. (2001) |
| *Dendropsophus triangulum* | Brazil: Acre: Ilha Xiborena: Lago Catalão | AY843680 | AY843680 | AY843680 | Faivovich et al. (2005) |
| *Dendropsophus walfordi* | Brazil | AY843683 | AY843683 | AY843683 | Faivovich et al. (2005) |
| **Outgroup** |  |  |  |  |  |
| *Lysapsus limellum* | Argentina: Corrientes: Bella Vista | AY843697 | AY843697 | AY843697 | Faivovich et al. (2005) |
| *Phyllodytes luteolous* | Brazil: Espírito Santo: Setiba, Guarapari | AY843721 | AY843721 | AY843721 | Faivovich et al. (2005) |
| *Pseudis minutus* | Argentina: Entre Rios: Departamento Islas del Ibicuy | AY843739 | AY843739 | AY843739 | Faivovich et al. (2005) |
| *Pseudis paradoxa* | Argentina: Corrientes: Departamento Bellavista | AY843740 | AY843740 | AY843740 | Faivovich et al. (2005) |
| *Scarthyla goinorum* | Brazil: Amazonas: Igarapé Nova Empresa | AY843752 | AY843752 | AY843752 | Faivovich et al. (2005) |
| *Scinax berthae* | Argentina: Buenos Aires, Atalaya | AY843754 | AY843754 | AY843754 | Faivovich et al. (2005) |
| *Scinax fuscovarius* | Argentina: Misiones, Guarani, San Vicente | AY843758 | AY843758 | AY843758 | Faivovich et al. (2005) |
| *Sphaenorhynchus dorisae* | Brazil: Amazonas: Manaus: Lago Janauri | AY843766 | AY843766 | AY843766 | Faivovich et al. (2005) |
| *Sphaenorhynchus lacteus* | Peru: Madre de Dios: Tambopata Reserve | AY549367 | AY549367 | AY549367 | Faivovich et al. (2005) |
| *Sphaenorhynchus orophilus* | Brazil: São Paulo: Salesópolis | DQ380388 | - | - | Wiens et al. (2006) |
| *Xenohyla truncata* | Brazil: Rio de Janeiro: Restinga de Marica | AY843775 | AY843775 | AY843775 | Faivovich et al. (2005) |

^1^= different vouchers of the 12S and 16S.

**References**

Chek AA, Lougheed SC, Bogart JP, Boag PT. Perception and history: molecular phylogeny of a diverse group of Neotropical frogs, the 30-chromosome *Hyla* (Anura: Hylidae). Molecular Phylogenetics and Evolution. 2001;18: 370–385.

Faivovich J, Haddad CFB, Garcia PCA, Frost DR, Campbell JA, Wheeler WC. Systematic review of the frog family Hylidae, with special reference to Hylinae: phylogenetic analysis and taxonomic revision. Bulletin of the American Museum of Natural History. 2005;294: 1–240.

Ferreira RB, Faivovich J, Beard KH, Pombal JPJr. The First Bromeligenous Species of *Dendropsophus* (Anura: Hylidae) from Brazil's Atlantic Forest. PLoS ONE. 2015;10: E0142893.

Fouquet A, Orrico VGD, Ernst R, Blanc M, Martinez Q, Vacher J-P, Rodrigues M, Ouboter P, Jairam R, Ron S. A new *Dendropsophus* Fitzinger, 1843 (Anura: Hylidae) of the parviceps group from the lowlands of the Guiana Shield. Zootaxa. 2015;4052: 39–64.

Jungfer K, Reichle S, Piskurek O. Description of a new cryptic southwestern Amazonian species of leaf-gluing treefrog, genus *Dendropsophus* (Amphibia: Anura: Hylidae). Salamandra. 2010;46: 204–213.

Motta AP, Castroviejo-Fisher S, Venegas PJ, Orrico VGD, Padial JM. A new species of the *Dendropsophus parviceps* group from the western Amazon Basin (Amphibia: Anura: Hylidae). Zootaxa. 2012;3249: 18–30.

Peloso PL V, Orrico VGD, Haddad CFB, Lima-Filho GR, Sturaro, MJ. A new species of Clown Tree Frog, *Dendropsophus leucophyllatus*species group, from Amazonia (Anura, Hylidae). South American Journal of Herpetology. 2016;11: 66–80.

Rivera-Correa M, Orrico VGD. Description and phylogenetic relationships of a new species of treefrog of the *Dendropsophus leucophyllatus* group (Anura: Hylidae) from the Amazon basin of Colombia and with an exceptional color pattern. Zootaxa. 2013;3686: 447–460.

Wiens JJ, Graham CH, Moen DS, Smith SA, Reeder TW. Evolutionary and ecological causes of the latitudinal diversity gradient in hylid frogs: treefrog trees unearth the roots of high tropical diversity. The American Naturalis. 2006;168: 579–596.

Wiens JJ, Reeder TW, Fetzner JW Jr, Parkinson CL, Duellman WE. Hylid frog phylogeny and sampling strategies for speciose clades. Systematic Biology. 2005;54: 778–807.
