# Supplementary material for: The 100th: An appealing new species of Dendropsophus (Amphibia: Anura: Hylidae) from northeastern Brazil
Source: PLoS One. 2017 Mar 8;12(3):e0171678. doi: 10.1371/journal.pone.0171678 (PMC5342187; doi:10.1371/journal.pone.0171678)
Supplement: S3 Appendix — (DOC) [file pone.0171678.s003.doc]

**S3 Appendix. Measurements morphometric of each individual analyzed of the type-series of *Dendropsophus nekronastes* sp. nov.**

| **-** | **SVL** | **HL** | **HW** | **ED** | **TYD** | **IND** | **IOD** | **ENN** | **NSD** | **THL** | **TL** | **FL** | **TAL** | **4TD** | **DF3** | **HAL** | **FOL** | **Sex** |
| --- | --- | --- | --- | --- | --- | --- | --- | --- | --- | --- | --- | --- | --- | --- | --- | --- | --- | --- |
| **MZUESC 10174** | 24.1 | 7.5 | 7.8 | 3 | 1.1 | 2.1 | 3.7 | 2 | 1.3 | 13.5 | 14.1 | 12.2 | 7.4 | 1.5 | 1.4 | 8.1 | 4.2 | Male |
| **MZUESC 10175** | 26.6 | 9 | 8.7 | 2.9 | 1.3 | 2.5 | 4 | 2.8 | 1.4 | 14.9 | 15.5 | 13.0 | 7.7 | 1.4 | 1.4 | 8.8 | 4.3 | Male |
| **MZUESC 10176** | 28.5 | 9.1 | 9.3 | 3.4 | 1.4 | 2.3 | 4 | 2.7 | 1.3 | 14.9 | 15.65 | 13.9 | 8.3 | 1.5 | 1.7 | 9.4 | 5.1 | Male |
| **MZUESC 10177** | 27.4 | 8.6 | 8.7 | 3.4 | 1.4 | 2.3 | 4 | 2.4 | 1.4 | 13.7 | 14.6 | 12.6 | 7.4 | 1.5 | 1.7 | 8.3 | 4.6 | Male |
| **MZUESC 10178** | 28.5 | 9.3 | 9.5 | 3.5 | 1.6 | 2.5 | **4.2** | 2.6 | 1.4 | 14.8 | 16.1 | 13.8 | 8.0 | 1.7 | 1.8 | 9.3 | 4.8 | Male |
| **MZUESC 10179** | 27.4 | 8.9 | 9.1 | 3.3 | 1.4 | 2.4 | 3.7 | 2.7 | 1.4 | 14.3 | 15.1 | 12.6 | 7.5 | 1.3 | 1.3 | 8.4 | 5.1 | Male |
| **MZUESC 10180** | 27.9 | 9 | 9.3 | 3.2 | 1.4 | 2.5 | 3.7 | 2.7 | 1.4 | 14.4 | 15.5 | 13.5 | 7.2 | 1.4 | 1.6 | 8.7 | 5.4 | Male |
| **MZUESC 10181** | 26.8 | 8.7 | 8.8 | 3.5 | 1.4 | 2.3 | 3.5 | 2.6 | 1.3 | 13.7 | 14.5 | 12.3 | 7.5 | 1.2 | 1.4 | 8.0 | 4.3 | Male |
| **MZUESC 10182** | 26.2 | 8.7 | 8.8 | 3.3 | 1.3 | 2.2 | 3.6 | 2.7 | 1.1 | 13.85 | 14.65 | 12.8 | 7.0 | 1.1 | 1.3 | 8.1 | 5.2 | Male |
| **MZUESC 10183** | 27.3 | 9.1 | 9.1 | 3.5 | 1.5 | 3 | 3.7 | 2.6 | 1.3 | 14.65 | 15.5 | 13.3 | 8.0 | 1.4 | 1.5 | 8.7 | 5.1 | Male |
| **MZUESC 10184** | 28.9 | 9 | 9.1 | 3.3 | 1.5 | 2.6 | 3.7 | 2.6 | 1.4 | 14.3 | 15.5 | 13.8 | 8.4 | 1.5 | 1.5 | 9.0 | 5.4 | Male |
| **MZUESC 10185** | 31.8 | 9.35 | 9.7 | 3.1 | 1.5 | 2.6 | **4.3** | 3.2 | 1.5 | 15.8 | 14.65 | 15.1 | 8.0 | 1.5 | 1.6 | 9.9 | 5.5 | Female |
| **MZUESC 10221** | 28 | 8.8 | 8.7 | 3.1 | 1.2 | 2.5 | 3.7 | 2.5 | 1.4 | 14.1 | 15 | 13.6 | 7.5 | 1.2 | 1.4 | 9.0 | 4.5 | Male |
| **MZUESC 10222** | 28 | 8.9 | 9.0 | 3.3 | 1.3 | 2.5 | 4.1 | 2.7 | 1.3 | 15 | 15.6 | 13.2 | 7.8 | 1.1 | 1.1 | 8.8 | 5.5 | Male |
| **MZUESC 10223** | 26.9 | 8.5 | 8.8 | 2.9 | 1.3 | 2.4 | 3.7 | 2.7 | 1.1 | 14.2 | 15.4 | 12.6 | 8.0 | 1.2 | 1.5 | 8.7 | 4.8 | Male |
| **MZUESC 9979** | 35.4 | 11.2 | 10.7 | 3.8 | 1.7 | 3.8 | 4.2 | 3 | 1.6 | 18.35 | 19.5 | 17.7 | 9.7 | 2.1 | 2.4 | 11.6 | 6.5 | Female |
| **MZUESC 9980** | 25.6 | 8.55 | 9.0 | 3.3 | 1.4 | 2.3 | 3.5 | 2.5 | 1.2 | 13.8 | 14.7 | 12.0 | 7.7 | 1.3 | 1.4 | 8.0 | 5.1 | Male |
| **MZUESC 9981** | 28.4 | 8.8 | 9.3 | 3.7 | 1.3 | 2.4 | 4 | 2.3 | 1.3 | 14.6 | 15.7 | 13.5 | 8.1 | 1.5 | 1.5 | 8.6 | 5.1 | Male |
| **MZUESC 9982** | 27.75 | 8.5 | 9.1 | 3.3 | 1.5 | 2.4 | 3.7 | 2.3 | 1.2 | 14 | 14.7 | 13.5 | 7.7 | 1.6 | 1.7 | 9.4 | 4.5 | Male |
| **MZUESC 9983** | 27.7 | 8.3 | 8.7 | 3.3 | 1.2 | 2.3 | 3.7 | 2.5 | 1.2 | 14.45 | 15.55 | 13.5 | 7.8 | 1.5 | 1.5 | 8.3 | 5.2 | Male |
| **MZUESC 9984** | 26.4 | 8.55 | 9.0 | 3.5 | 1.6 | 2.2 | 3.7 | 2.5 | 1.1 | 13.5 | 14.25 | 12.6 | 7.5 | 1.3 | 1.5 | 8.0 | 5.1 | Male |
| **MZUESC 9985** | 27.2 | 8.7 | 8.7 | 3.5 | 1.4 | 2.2 | 3.5 | 2.3 | 1.2 | 13.9 | 14.8 | 12.8 | 7.8 | 1.4 | 1.6 | 8.7 | 4.3 | Male |
| **MZUESC 9986** | 26.5 | 8.7 | 9.1 | 3.5 | 1.1 | 2.4 | 3.6 | 2.5 | 1.4 | 13.8 | 15 | 12.8 | 7.5 | 1.4 | 1.4 | 8.6 | 5.1 | Male |
| **MZUESC 9987** | 27.55 | 8.6 | 8.8 | 3.4 | 1.5 | 2 | 3.7 | 2.6 | 1 | 14 | 14.8 | 13.6 | 7.8 | 1.5 | 1.7 | 8.4 | 5.2 | Male |
| **MZUESC 9988** | 27.2 | 8.5 | 9.0 | 2.8 | 1.3 | 2.2 | 3.9 | 2.7 | 1.3 | 12.7 | 14.1 | 12.5 | 7.8 | 1.2 | 1.4 | 8.4 | 5.1 | Male |
| **MZUESC 9989** | 28.3 | 9 | 9.0 | 3.7 | 1.3 | 2.2 | 3.8 | 2.7 | 1.1 | 14.4 | 15.5 | 13.3 | 8.1 | 1.6 | 1.8 | 9.0 | 4.5 | Male |
